# Supplementary material for: Longer Participation in the Special Supplemental Nutrition Program for Women, Infants, and Children Is Not Associated with Reduced Sugar-Sweetened Beverage Intake among Black Participants
Source: Nutrients. 2022 Feb 28;14(5):1048. doi: 10.3390/nu14051048 (PMC8912710; doi:10.3390/nu14051048)
Supplement: Supplementary file 1 [file nutrients-14-01048-s001.zip › nutrients-1563750-supplementary.pdf]

Supplemental Table S1 Relative rate of daily intake of total SSB, fruit-flavored SSB, and water for non-White compared to White children at 1, 5 and 10 years of family WIC participation (n=11,482).

		1 years [c]	5 years [d]	10 years [e]
Total SSB [a]				
	Asian	0.81 (0.57, 1.13)	**0.74 (0.59, 0.92)**	0.77 (0.53, 1.13)
	Black	1.05 (0.79, 1.39)	**1.26 (1.08, 1.48)**	**1.52 (1.23, 1.87)**
	Hispanic, SP	0.86 (0.68, 1.09)	**0.87 (0.76, 0.99)**	0.93 (0.78, 1.10)
	Hispanic, EN	**1.31 (1.02, 1.69)**	1.04 (0.92, 1.18)	1.01 (0.85, 1.20)
	White	1.00 (ref)	1.00 (ref)	1.00 (ref)
Fruit-flavored SSB [a]				
	Asian	0.91 (0.59, 1.40)	0.83 (0.62, 1.10)	0.92 (0.57, 1.49)
	Black	1.28 (0.90, 1.81)	**1.73 (1.44, 2.08)**	**2.10 (1.63, 2.70)**
	Hispanic, SP	0.96 (0.71, 1.31)	0.97 (0.82, 1.13)	1.05 (0.84, 1.30)
	Hispanic, EN	1.33 (0.96, 1.83)	1.09 (0.93, 1.27)	1.11 (0.89, 1.37)
	White	1.00 (ref)	1.00 (ref)	1.00 (ref)
Water [b]				
	Asian	1.01 (0.92, 1.11)	1.02 (0.95, 1.10)	0.97 (0.86, 1.10)
	Black	**0.86 (0.79, 0.94)**	**0.87 (0.82, 0.92)**	**0.86 (0.80, 0.94)**
	Hispanic, SP	**0.92 (0.85, 0.99)**	**0.95 (0.91, 1.00)**	0.95 (0.90, 1.01)
	Hispanic, EN	1.00 (0.93, 1.09)	1.00 (0.96, 1.05)	0.98 (0.93, 1.04)
	White	1.00 (ref)	1.00 (ref)	1.00 (ref)

CI=confidence interval; EN=English-speaking; IRR=incidence rate ratio; SP=Spanish-speaking; SSB=sugar-sweetened beverage; WIC=the Special Supplemental Nutrition program for Women, Infants and Children; yr=years

[a] Estimates are from negative binomial regression models including terms for the child's age, race/ethnicity and survey year; maternal education, age, and BMI; household size, income, food security status, SNAP participation; family duration on WIC (linear and quadratic); and the interaction of family duration on WIC and child race/ethnicity.

[b] Estimates are from Poisson regression models including terms for the child's age, race/ethnicity and survey year; maternal education, age, and BMI; household size, income, food security status, SNAP participation; family duration on WIC (linear and quadratic); and the interaction of family duration on WIC and child race/ethnicity.

[c] IRR (95% CI) for daily servings of each beverage comparing children of different racial/ethnic groups from families with 1 years of WIC participation.

[d] IRR (95% CI) for daily servings of each beverage comparing children of different racial/ethnic groups from families with 5 years of WIC participation..

[e] IRR (95% CI) for daily servings of each beverage comparing children of different racial/ethnic groups from families with 10 years of WIC participation.
